# Molecular Mechanisms of Carcinogenesis in Pediatric Airways Tumors

**DOI:** 10.3390/ijms24032195

**Published:** 2023-01-22

**Authors:** Davide Soloperto, Sandra Gazzini, Raffaele Cerullo

**Affiliations:** Division of Otolaryngology, Head and Neck Surgery Department, University Hospital of Verona, Piazzale L.A. Scuro 10, 37134 Verona, Italy

**Keywords:** pediatric airways tumors, pediatric laryngeal tumors, tumor biology, tumorigenesis, molecular mechanisms, subglottic hemangioma, recurrent respiratory papillomatosis, rhabdomyosarcoma, mucoepidermoid carcinoma, children

## Abstract

Primary tumors of the airways in the pediatric population are very rare entities. For this reason, little is known about the pathogenesis of these neoplasms. Understanding the biology has different practical implications: for example, it could help in the differential diagnosis, have a prognostic significance, or may lead to the development of a targeted therapy. The aim of this article is to present the current knowledge about pediatric airways tumors, focusing on the molecular mechanisms that cause the onset and progression of these neoplasms. After a brief introduction of epidemiology and clinical presentation, the tumorigenesis of the most frequent pediatric airways tumors will be described: Juvenile-onset recurrent respiratory papillomatosis (JORRP), Subglottic Hemangiona (SH), Rhabdomyosarcoma (RMS), and Mucoepidermoid carcinoma (MEC).

## 1. General Considerations

The airway, or respiratory tract, is a complex anatomical district that includes the organs that allow airflow during ventilation. It is generally subdivided into upper and lower airways, which are in anatomical continuity between each other. The subdivision is mainly based on clinical aspects related to the spread of infectious processes and the mode of embryonic development. The upper airways include the nose, nasal cavity, pharynx, and larynx; the lower airways comprise the trachea, the bronchial tree, and the lungs.

Primary airway tumors are very rare in the pediatric population. Consequently, this kind of tumors usually attracts little research attention and the literature mainly consists of case reports or case series. Nevertheless, some steps toward the understanding of the biology of these tumors have been made. The scope of this review is to summarize the current literature on this topic, focusing on the larynx and lower airways.

## 2. Introduction to Pediatric Laryngeal Tumors

Pediatric tumors of the larynx are uncommon diseases and benign tumors represent the vast majority of them (98%) [1]. Among benign pediatric lesions, the most frequent entities are recurrent respiratory papillomatosis (RRP) and subglottic hemangioma (SH), with a reported incidence in Western countries of 3–4 per 100,000 and 2 per 100,000 live births, respectively [2,3]. Less frequently encountered entities are schwannoma, paraganglioma, chondroma, lipoma, and pleomorphic adenoma. Malignant laryngeal tumors in children are extremely rare (2% of laryngeal tumors), with squamous cell carcinoma and sarcoma reported as the most common histological types [4]. Among sarcomas, rhabdomyosarcoma is the most frequent tumor [5]. The clinical symptoms are usually inspiratory stridor or dysphonia/dyspnea, and the mode of presentation depends on tumor size and location [1]. The diagnosis includes fiberoptic laryngoscopy and, when the clinical suspicion is high, direct laryngoscopy under general anesthesia can show tumor extension and permit biopsy, if needed. Imaging (CT and/or MRI) can help to better define deep extension and relationships with critical structures. The treatment mainly depends on the histological type and extent of disease and comprises surgery, radiotherapy, and/or chemotherapy.

**Figure 1:**
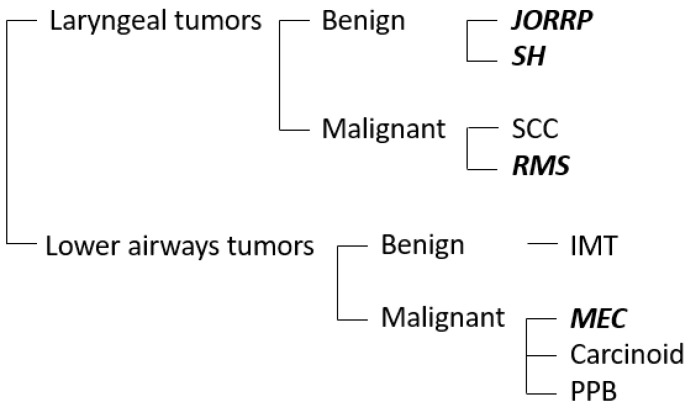
The chart represents the most frequent benign and malignant airways tumors in the pediatric population. The neoplasms discussed in this review are highlighted in bold. JORRP, juvenile-onset recurrent respiratory papillomatosis; SH, subglottic hemangioma; SCC, squamous cell carcinoma; RMS, rhabdomyosarcoma; IMT, inflammatory myofibroblastic tumor; MEC, mucoepidermoid carcinoma; PPB, pleuropulmonary blastoma.

Given the rarity of these lesions, little is known about the precise molecular mechanisms underlying each of these tumors. The existing literature mainly focuses on the most frequent entities (RRP and SH), which will be thoroughly discussed below. Then, a brief review of the biology of malignant tumors is made, focusing on rhabdomyosarcoma (Figure 1).

## 3. Benign Laryngeal Tumors

### 3.1. Juvenile-Onset Recurrent Respiratory Papillomatosis

Juvenile-onset recurrent respiratory papillomatosis (JORRP) is a rare chronic disease characterized by multiple epithelial papillomas along the respiratory tract. JORRP is the most common laryngeal neoplasm in children and tends to be more aggressive than its adult counterpart [6,7]. The disease is typically diagnosed at the age of 2–3 years and there is a strong etiological association with human papilloma virus (HPV) infection, especially HPV 6 and HPV 11 subtypes, with higher prevalence of HPV6 [8]. HPV infection in affected newborns probably occurs when the fetus passes through an infected birth canal; however other ways of transmission have been proposed, such as vertical transmission in utero and horizontal transmission via the child’s environment [9,10]. JORRP has a strong negative impact on patients’ quality of life, with more than four average surgeries performed in the first year following diagnosis [11]. In fact, the main treatment is surgical removal, but papillomas have high rates of postoperative recurrence. In order to reduce the recurrence rate, adjuvant therapies have been introduced in the case of aggressive disease, such as intralesional cidofovir, anti-angiogenic factors and interferons, showing variable effectiveness [1].

With regards to the molecular mechanisms underlying this kind of tumor, many studies have been published over the years, but some aspects still remain unclear. As previously mentioned, JORRP is usually caused by low-risk HPV types 6 or 11, which are also the most common cause of anogenital warts. However, only 0.7% of infants exposed to maternal anogenital warts develops recurrent respiratory papillomas (RRP), suggesting other factors to be involved in the pathogenesis [12,13]. HPV infects the basal layer of the epithelium of the respiratory and aerodigestive tracts through minor excoriation [14]. Then, by activating the epidermal growth factor receptor (EGFR) pathway and inhibiting oncosuppressors, it stimulates cellular proliferation [15]. This results in typical cauliflower-like exophytic lesions, called papillomas. In JORRP, the lesions are frequently located in transitional zones between the squamous epithelium and the ciliated columnar epithelium and the larynx is almost invariably involved. The spread to the distal airways occurs in only 2–5% of patients, and pulmonary involvement is seen in only 1% of cases [16].

High-risk viral oncoproteins, E6 and E7, have been extensively studied. E6 targets p53 and sends it to degradation, whereas E7 targets the oncosuppressor Rb, thereby altering its DNA repair pathways [17,18]. These mechanisms play a significant role in tumorigenesis caused by high-risk HPV, but in low-risk HPV, E6 and E7 proteins show lower affinity to their targets and less transforming activity [13,19]. As a result, in JORRP patients HPV-DNA is often episomal and not integrated into the host genome [20]. E6/E7 have been shown to be involved in the viral life cycle, interacting with the host immune system, and altering the immune response. They mainly cause an imbalance in the adaptive immune response by interacting with different immune pathways [21]. Such an imbalance is caused by interfering with the secretion of several proinflammatory cytokines, interleukins (ILs) and interferons (IFNs). Specifically, E6 inhibits the expression of IL-2 and IL-18, two pro-inflammatory cytokines that are directly involved in T-helper 1 (Th1) immune response to HPV, thereby reducing the cytotoxic T-cell activity in JORRP patients [22]. E7 interacts with Transporter associated with antigen processing 1 (TAP-1), a protein that carries peptides into the endoplasmic reticulum for presentation by major histocompatibility complex (MHC) class I [23]. The reduced expression of antigen by MHC-I results in less activity of cluster of differentiation 8-positive (CD8+) cytotoxic T-lymphocytes. This unfavorable environment for CD8+ T-cells maturation causes a shift toward a Th2-like response, blocking specific Th1-cytotoxic response against HPV and enhancing immune tolerance to the virus [24]. This shift reflects in the higher levels of Th2 cytokines, such as IL-4 and IL-10, as well as transforming growth factor-beta (TGF-β), found in both peripheral blood and tumor tissue in JORRP patients [25,26]. Other presumed mechanisms involve the deficiency of innate immune response. Studies have found a dysfunction of macrophages and Langerhans cells in the papilloma microenvironment, which may secrete chemokines (C-C motif) ligand (CCLs), such as CCL17 and CCL18, in response to the Th2-like interleukins [27]. Such chemokines can prompt naïve CD4+ T cells to become memory Th2-like T cells and regulatory T cells (Tregs) [21]. Treg cells are essential for immune tolerance and higher levels of a particular subtype of these lymphocytes (CD4+CD25+CD127low/−Foxp3+) have been found in papillomatous tissue [28]. These mechanisms may be responsible for reduced IFN-γ in JORRP patients, that is normally secreted by activated T cells and macrophages and supports Th1 response. Another alternative mechanism could be direct inhibition of IFN-γ production by E6 protein [22]. These data indicate that the alterations in immune function induced by HPV play a central role in the pathogenesis of JORRP. Moreover, the critical period for the development of the immune system in infants occurs between 4 and 6 years of age, mirroring the first age distribution peak of JORRP [21].

The disease course of JORRP can be extremely variable among individuals. According to Doyle et al., criteria for aggressiveness are total number of surgeries ≥10, average number of operations per year ≥3, and distal spread to lower airways [29]. Classically, younger age at diagnosis and infection with HPV11 have been associated with a more aggressive course [7]. The correlation between younger age and aggressive disease could be explained by the predisposition of the immune system of younger infants to tolerance and/or by reduced airway size. On the other hand, the major oncogenic potential of HPV11 compared to HPV6 could be related to more oncogenic targets of HPV11 E6 and E7 or to the different viral life cycle [21,30]. However, after accounting for confounding between HPV11 and young age, HPV type was minimally associated with aggressiveness in a multicenter study. Therefore, younger age at diagnosis (0–5 years) appears to be a more powerful indicator of aggressive disease [7].

One of the main issues when dealing with JORRP, as the name suggests, is the high rate of recurrence. The pathogenesis of recurrence is still unclear. A study on 70 patients reported that in the vast majority of cases, there is a complete genetic identity between genomes of first isolated HPV and a second isolate obtained up to 22 years later [31]. This leads to the hypothesis that recurrence may occur due to reactivation of latent HPV in adjacent tissue, which acts as viral reservoir [21]. Some authors believe that a trigger of HPV reactivation could be local inflammation due to surgical removal of papillomas, while others have showed a link to gastroesophageal reflux disease [32,33]. In this scenario, adjuvant treatment with HPV vaccine after surgery has been suggested in recent years to prevent recurrences. HPV inactivated vaccine (Gardasil) may be quadrivalent (against HPV subtypes 6, 11, 16 and 18) or nonavalent (against HPV subtypes 6, 11, 16, 18, 31, 33, 45, 52 and 58). (2). The vaccines are prepared using recombinant technology to develop virus-like particles derived from L1 viral capsid and they elicit a more robust adaptive immune response [34]. A systematic review has showed that 9 of 12 studies reported decreased disease recurrence or increased intersurgical interval after adjuvant HPV vaccination [35]. Despite the promising results, further long-term research is required to assess the role of HPV vaccination in both prophylactic and therapeutic setting.

JORRP is a benign disease but a low rate of malignant transformation (1–2%) has been reported [36]. The only histological type that has been directly associated with RRP, is squamous cell carcinoma, usually well-differentiated, which can affect the larynx, the trachea, and the lungs [37,38]. The malignant transformation is far more common in adults, with irradiation and smoking indicated as risk factors [39]. The molecular basis of carcinogenesis from RRP is still poorly understood. However, some studies have shown that various proteins involved in tumorigenesis are differentially expressed in papillomas and associated with malignancy, such as chemokine (C-X-C motif) ligand (CXCL) 1, CXCL6, CXCL8, and vascular endothelial growth factor (VEGF)-A. These mediators promote angiogenesis, which plays a key role in malignant transformation [40,41]. The important role of angiogenesis in JORRP pathogenesis has led Bevacizumab (anti-VEGF-A humanized monoclonal antibody) to be used as adjuvant therapy by some authors [42]. HPV11 may also have an important tumorigenic role in some patients without risk factors for malignancy [43]. Given the progressively increased expression of p53 and Rb along with a reduced expression of p21WAF1 protein (involved in apoptotic response to DNA damage) during the progression to carcinoma, it has been hypothesized that mutations in regulatory regions of viral oncoproteins or integration of HPV into the host cell genome may be key events [20,43].

In conclusion, JORRP is a refractory disease that is incurable with current treatment modalities, especially when it fulfils the criteria for aggressiveness [21]. Research about this topic throughout the years has introduced adjuvant therapies in the clinical practice, with long-term effects that have still to be clarified. Further research about the molecular biology of this tumor could lead to introduce targeted therapy directed to the papilloma microenvironment. This could help to manage RRP patients more effectively in the next future.

### 3.2. Subglottic Hemangioma

Infantile hemangioma is a benign proliferative vascular tumor. The prevalence ranges between 4 and 10% and it is more frequent in females, Caucasians, and premature newborns. It is not present at birth, although precursor lesions such as telangiectasias or erythematous macules can be visible in 30–50% of cases [44]. Subglottic localization of hemangioma is rare but potentially life-threatening, because of the small caliber of the infant’s airway, that can be easily obstructed by the neoplasm during the rapid growth phase [45].

Patients with subglottic hemangiomas (SH) are usually asymptomatic at birth and during the first weeks of life; later, they can show respiratory distress (both inspiratory and expiratory stridor) and barking cough that can be initially misdiagnosed as croup. They usually do not show dysphonia or dysphagia, but they might have feeding difficulties because they struggle to breath and suck at the same time [46].

SH can be associated with cutaneous hemangiomas in the “beard distribution”, involving preauricular skin, anterior neck, or lower lip area [46].

Although the etiology is still not completely defined, recent data show the expression of primitive markers in SH, suggesting an embryonic developmental anomaly of hemogenic endothelium that possesses the ability of neuronal, mesenchymal, endothelial, and hemopoietic differentiation [47]. In particular, recent studies have demonstrated the expression of markers such as hCG (human chorionic gonadotropin) and hPL (human placental lactogen), but not CK7 (cytokeratin-7) and HLA-G (human leukocyte antigen G), suggesting that SH could derive from placental chorionic villous mesenchymal core cells, that have angiogenic capability, migrating via umbilical vein into the fetal circulation [48]. It is not clear if SH results from a genetic mutation or it is consequence of a post-zygomatic somatic mutation in a progenitor cell. Familial hemangiomas are rare and are characterized by autosomal dominant inheritance and have been linked to chromosome 5q31–33. Sporadic hemangiomas seem to be caused by loss of heterozygosity of chromosome 5q as well, suggesting that hemangioma could be the result of the loss of a specific gene that has a role in tumor suppression [49,50].

In the evolution of SH, we can recognize a first phase of rapid growth, called proliferative phase, from 3 to 10 months of age, followed by the involutional phase, with regression within 5–10 years of age [51]. Histological analysis shows that during the first phase the tumor is highly cellular, rich in small vascular channels, with immature endothelial and interstitial cells, and few connective tissue; conversely, in the second phase fewer interstitial cells are found and deposition of extracellular matrix occurs, building up around more prominent and mature blood vessels [52].

In 2008, Léauté-Labrèze et al. published a letter to the editor, presenting the promising results of the use of propranolol, a nonselective beta-adrenergic antagonist, in the treatment of hemangioma in 11 children [53]. After that first serendipitous utilization, propranolol was introduced in clinical practice as first line treatment for cutaneous and subglottic hemangiomas, giving excellent results [54]. Although the pathophysiology of SH is still poorly understood, studies about propranolol’s action have led to new knowledge about the mechanisms of development of hemangiomas as well.

The typical evolution of subglottic hemangioma seems to be determined by imbalance of angiogenic factors, with predominance of VEGF, basic growth factor of fibroblast (bFGF) and matrix metalloproteinases (MMPs) 2 and 9 during the proliferation phase, that provoke a rapid vascular growth. The involution phase begins when the levels of these factors decrease and those of antiangiogenic factors, such as tissue inhibitors of metalloproteinases (TIMP), increase [44].

Pathological angiogenesis is composed of four steps: 1. Hypoxemia, that stimulates the release of mitogenic factors; 2. induction of proliferation of vascular endothelial cells; 3. migration of endothelial cells following chemotactic stimulus; 4. degradation of the extracellular matrix around the vessels, to permit endothelial cell invasion of new territories [55]. The main actor of this process is VEGF, which can induce endothelial progenitor cells chemiotaxis, proliferation, and differentiation in endothelial cells, causing the formation of new vessels [56,57]. The elevated levels of VEGF found in SH tissues are induced by hypoxemia. In fact, oxygen deficiency leads to an increase in intracellular concentration of hypoxia-inducible factor 1a (HIF-1a), that induces the transcription of many genes involved in vasculogenesis, in particular VEGF gene. This protein, secreted from the cells, diffuses into the surrounding tissues, inducing endothelial cells proliferation [58,59]. Considering the epidemiology (the tumor is more frequent in prematurity, multiple gestations, advanced maternal age, low birth weight), it is hypothesized a possible role of placenta malfunction in the pathogenesis of hemangiomas, that results in tissue hypoxemia in infants [50,60]. Up-regulation of VEGF can also occur through the action of noradrenaline. The stimulation of beta-adrenoreceptor increases cAMP levels, which activates protein kinase A (PKA), which in turn activates the cytoplasmatic tyrosine kinase Src [58,61]. This kinase induces intracellular transcription of VEGF. Immunohistochemical findings of elevated expression of angiotensin receptor 2 in proliferating hemangioma led to the hypothesis that the renin-angiotensin system may contribute to the tumoral pathophysiology. In fact, angiotensin 2 seems to cause secretion of VEGF and proliferation of vascular progenitor cells expressing CD34, preventing their final differentiation [50,62,63].

Other important factors in the pathophysiology of SH are metalloproteinases MMP-2 and MMP-9, membrane-anchored proteinases that play a key role in degradation and remodeling of extracellular matrix around vessels, allowing migration of endothelial cells, tubulogenesis and the formation of new vessels. By degrading the basement membrane and extracellular matrix, MMPs provide spaces for vasculogenesis; in addition, products of this degradation have chemotactic effect on endothelial cells. Expression of MMP-2 and MMP-9 is regulated via beta-adrenoceptors, likely through the action of VEGF [44,58,64]. Natural inhibitors of MMPs are tissue inhibitors of metalloproteinases (TIMP), that reduce extracellular matrix degradation. Under physiological conditions, MMPs and TIMPs are in dynamic equilibrium [64,65]. On the contrary, MMPs are overexpressed in the progression phase of SH, whereas TIMPs are increased in involution phase.

Although infantile hemangioma is a benign disease characterized by spontaneous regression, SH needs treatment in most cases, because it is potentially life-threatening due to the anatomical location. Nowadays, first-line treatment is propranolol. This drug, blocking b1- and b2-adrenoceptors, acts against SH on three different levels. First, it antagonizes adrenaline-mediated vasodilatation, causing reduction of blood flow within SH. Second, it inhibits angiogenesis, reducing the expression of VEGF, both blocking beta-adrenoreceptor (and consequently the cAMP-Src cascade) and the renin-angiotensin system. Third, it can disengage the inhibition of apoptosis caused by beta-adrenergic agonists (mediated by MAPK and caspase cascade pathway), resulting in an increased apoptosis rate [58].

In summary, almost 15 years after the introduction of propranolol in the clinical practice, some steps have been made toward the understanding of the pathophysiology of SH. Nevertheless, some aspects are yet to be elucidated.

## 4. Malignant Laryngeal Tumors

### Rhabdomyosarcoma

Rhabdomyosarcoma (RMS) is an aggressive soft tissue tumor, which develops from the embryonic mesenchyme of skeletal muscle lineage. In fact, it expresses skeletal muscle markers such as myosin, desmin, and myoglobin [66]. RMS is the most common sarcoma in the pediatric population, accounting for around 5% of all malignant tumors in children [67]. It can involve different body areas, but almost half of the cases occurs in the head and neck district. However, RMS is rarely located in the larynx [68]. From a histological point of view, RMS can be divided into four subtypes: embryonal (ERMS, accounting for 60–70% of cases), alveolar (ARMS, 20%), pleomorphic, and spindle cells RMS [69]. ERMS is more frequent in children younger than 10 years and is characterized by favorable prognosis. It usually arises in muscle tissues of head and neck. On the other hand, ARMS is more frequent in adolescent, has a worse prognosis, and typically develops in the extremities and trunk [66].

**Figure 2:**
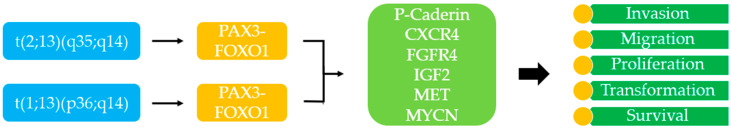
The fusion protein PAX3/7-FOXO1 is a transcriptional factor that is supposed to have a role in stopping skeletal muscle differentiation and facilitating aberrant cell division, resulting in tumor development. In the figure, some transcriptional targets of PAX3/7-FOXO1 are shown, which have a role in migration, proliferation, transformation, and survival of tumoral cells in RMS.

From a genetic and cytogenetic point of view, ARMS is frequently characterized by the chromosomal translocation t(2;13)(q35;q14), resulting in the expression of the oncogenic fusion protein PAX3-FOXO1 (paired box protein 3—forkhead boxprotein O1). Another translocation described in the literature is t(1;13)(p36;q14) that results in the fusion protein PAX7-FOXO1 [69]. The fusion protein PAX3/7-FOXO1 is a transcriptional factor that is supposed to have a role in stopping skeletal muscle differentiation and facilitating aberrant cell division, resulting in tumor development [66]. Many different transcriptional targets of PAX3/7-FOXO1 have been identified, such as genes for P-Caderin, CXCR4, FGFR4, IGF2, MET, MYCN. The products of this transcription have a key role in determining migration, proliferation, malignant transformation, and survival of tumoral cells (Figure 2) [66,70]. PAX3/7-FOXO1 expression is supposed to be one of the earliest events in ARMS tumorigenesis, but in vitro experiments have demonstrated that this factor alone is not sufficient to induce alterations in normal cells. This finding has led to the hypothesis that probably other genetic alterations are implicated in the tumorigenesis.

Unlike ARMS, ERMS does not show any specific chromosomal translocations. In contrast, it is characterized by genomic instability and chromosomal imbalance, which result in extra copies of chromosomes 2, 7, 8, 12, 13, and 20 [69,71].

RMS can also be associated with loss of heterozygosity or loss of imprinting at 11p15.5, region that contains several genes. One of this is insulin-like growth factor 2 (IGF2), which is upregulated by changes in imprinting or loss of heterozygosity at this locus [66]. In fact, many studies report an overexpression of IGF2 in Rhabdomyosarcomas, that serves as an autocrine signal for cellular growth. IGF pathway has a critical role in carbohydrates metabolism and glucose control, and it consequently promotes cellular mitosis, differentiation, and growth. IGF2 binds to IGF receptor type 1 (IGF1R), determining its phosphorylation and activating an intracellular cascade that induces the kinases AKT and phosphatidyl inositol 3-kinase (PI3K), which are involved in signaling for cell proliferation and survival [72,73]. Although the abnormal expression of IGF2 is frequent in many types of malignancies and it seems to have a central role in the pathogenesis of RMS as well, the precise mechanism of tumorigenesis is still unclear [72]. However, IGF pathway-related proteins have not yet been implemented as cancer biomarkers due to contradictory findings.

Concerning RMS treatment, it is currently based on multiagent chemotherapy, combined to radiotherapy in most patients. Surgical removal is limited to selected cases [74]. Despite the important evolution in the treatment of RMS in the past decades, a targeted therapy is still missing since the pathogenesis has not been completely clarified.

## 5. Lower Airways Tumors

Primary tumors of the lower airways are very rare in the pediatric population, accounting for less than 1% of all pediatric malignancies [75]. In the lung, metastatic disease from a non-pulmonary neoplasm is much more frequent than a primary lung tumor, and the latter has an annual incidence of 0.049 per 100,000 persons [76]. Therefore, when a pulmonary mass is found, a careful work-up is recommended in order to rule out the presence of a primary tumor located elsewhere in the body. Primary tumors located along the tracheobronchial tree are even rarer entities. When considering primary lung tumors, malignant tumors are three-times more frequent than the benign ones [77]. The most common benign tumor of the lung in the pediatric population is inflammatory myofibroblastic tumor (IMT), whereas the most common primary malignancies are carcinoid tumor, pleuropulmonary blastoma (PPB) and mucoepidermoid carcinoma (MEC). With regard to primary tracheal and tracheobronchial tumors, the most frequent pediatric malignancy is the mucoepidermoid carcinoma [78].

Considering the rarity of these tumors and the paucity of studies investigating their molecular mechanisms of carcinogenesis, our discussion will be focused on mucoepidermoid carcinoma.

### Mucoepidermoid Carcinoma

Mucoepidermoid carcinoma (MEC) is a malignant salivary gland tumor, which originates from the minor salivary glands of the submucosa of the tracheobronchial tree. To date, almost 150 cases of pediatric MEC involving the tracheobronchial tree have been reported in the literature, whereas it accounts for 10% of all primary lung malignancies in children [78]. The clinical presentation of MEC is similar to bronchial asthma (wheezing, respiratory distress, persistent cough, recurrent pneumonia), as the most common location is the mainstem bronchus. Therefore, diagnostic delay is the rule [79,80]. Diagnostic work-up includes chest X-ray and chest CT scan as first tools and then bronchoscopy is usually performed, because the tumor typically grows into the lumen and biopsy can be performed for confirmatory diagnosis. The treatment is usually limited to surgical resection for low-grade and limited tumors, whereas locally advanced and high-grade MECs can be managed with a multimodal treatment, but robust evidence is lacking.

In recent years, basing on some works on the biology of MECs, targeted therapy has been suggested as a new option in therapeutic spectrum of this tumor. These studies arise from the previous discovery of the overexpression of epidermal growth factor receptor (EGFR), or mutation in its tyrosine kinase domain, in a large proportion of patients with non-small cell lung cancer (NSCLC). This results in sustained action of signal transduction pathways, leading to cell proliferation or anti-apoptosis. Thus, targeted therapy with tyrosine kinase inhibitors (TKIs), such as gefitinib and erlotinib, has been included in clinical practice with promising results for some patients with NSCLC harboring this kind of mutations [81,82]. Regarding MECs of the lower airways, mutations altering the tyrosine kinase domain of EGFR (e.g., L858R, L861Q) have been found in almost 20% of tumors, favoring activation or stabilization of the activated form of the receptor [83,84,85]. Interestingly, all the reported mutations have been found in the Asian population [85]. Few attempts with TKIs administration in patients with metastatic MECs have been made, showing various degrees of tumoral regression, even in pediatric patients [83,86,87]. However, no activating EGFR mutations were detected in MECs of patients who reportedly responded to the TKI therapy, suggesting that other mechanisms may be involved in this sensitivity to TKIs. Moreover, one study found that a pulmonary MEC cell line with wild-type EGFR showed more sensitivity to gefitinib when compared to NSCLC cell lines with wild-type EGFR [88]. Although these findings might be promising, the role of TKI therapy in metastatic MECs is still unclear.

An even more common finding is the t(11;19)(q21;p13) translocation, which occurs in 60–70% of MECs of the salivary glands and has been found also in lung MECs [89,90]. This translocation creates a fusion oncogene, CRTC1-MAML2 (CRTC1, CREB-regulated transcription coactivator 1, also known as MECT1, MEC translocated 1; MAML2, mastermind-like 2) that has been defined as the major oncogenic driver in MECs [91]. The oncogene encodes a fusion transcript, the chimera CRTC1-MAML2, which has been linked to both Notch and cAMP signaling pathways. Regarding the first pathway, a possible mechanism could be the transcription of the Notch target gene HES1 even in the absence of Notch-ligand, thereby acting on proliferation and differentiation [89]. However, recent studies focused mainly on the activation of a CREB (cAMP response element-binding protein)-dependent transcriptional program, with recruitment of p300/CBP into the CREB complex and transactivation of AP-1 and MYC, contributing to CREB-independent activities [91,92,93]. Another work showed that CRTC1-MAML2 induces IGF-1 (insulin-like growth factor 1) expression via PPARγ (peroxisome proliferator-activated receptor gamma) activation, thereby inducing cellular growth and survival [94]. Moreover, the prognostic value of t(11;19)(q21;p13) translocation has been investigated, with controversial results [95,96]. A recent meta-analysis showed that the detection of the CRTC1-MAML-2 translocation appears to be useful as a prognostic factor in salivary MEC, with reduced mortality in translocation-harboring patients, though with low level of evidence [97].

As successfully validated in other tumors (e.g., chronic myeloid leukemia), CRTC1-MAML2 could be a target for therapeutic intervention in the future. However, further studies are needed to introduce this kind of therapy in the clinical practice.

## 6. Conclusions

The understanding of cancer biology is continuously evolving throughout the years. The increasing amount of knowledge we are acquiring has led to the introduction of new therapies in the clinical practice and improvement in personalized treatment as well. Tumors of the airways represent a small subset of this research and airways tumors in pediatric patients represent an even smaller subset, given their extreme rarity. Nevertheless, the impact of these tumors on patients’ and parents’ quality of life is strongly negative and some efforts have been made in the last years to understand the biology of this kind of tumors. Our overview about these mechanisms can help to summarize the current evidence on this topic. However, since many aspects remain unclear, further research is crucial for better understanding the biology of pediatric tumors, especially in a poorly explored field such as the airways.

## Data Availability

Not applicable.
